# Total Variation Wavelet-Based Medical Image Denoising

**DOI:** 10.1155/IJBI/2006/89095

**Published:** 2006-08-06

**Authors:** Yang Wang, Haomin Zhou

**Affiliations:** School of Mathematics, Georgia Institute of Technology, Atlanta, GA 30332-0430, USA

## Abstract

We propose a denoising algorithm for medical images based on a combination of the total variation minimization scheme and the wavelet scheme. We show that our scheme offers effective noise removal in real noisy medical images while maintaining sharpness of objects. More importantly, this scheme allows us to implement an effective automatic stopping time criterion.


Hindawi Publishing Corporation
International Journal of Biomedical Imaging
Volume 2006, Article ID 89095, Pages 1–6
DOI 10.1155/IJBI/2006/89095

### Total Variation Wavelet-Based Medical Image Denoising

**Yang Wang and Haomin Zhou**

*School of Mathematics, Georgia Institute of Technology, Atlanta, GA 30332-0430, USA*

Received 30 January 2006; Revised 12 May 2006; Accepted 12 May 2006

We propose a denoising algorithm for medical images based on a combination of the total variation minimization scheme and the wavelet scheme. We show that our scheme offers effective noise removal in real noisy medical images while maintaining sharpness of objects. More importantly, this scheme allows us to implement an effective automatic stopping time criterion.

Copyright © 2006 Y. Wang and H. Zhou. This is an open access article distributed under the Creative Commons Attribution License, which permits unrestricted use, distribution, and reproduction in any medium, provided the original work is properly cited.

### 1. INTRODUCTION

The advent of digital imaging technologies such as MRI has revolutionized modern medicine. Today, many patients no longer need to go through invasive and often dangerous procedures to diagnose a wide variety of illnesses. With the widespread use of digital imaging in medicine today, the quality of digital medical images becomes an important issue. To achieve the best possible diagnoses it is important that medical images be sharp, clear, and free of noise and artifacts. While the technologies for acquiring digital medical images continue to improve, resulting in images of higher and higher resolution and quality, noise remains an issue for many medical images. Removing noise in these digital images remains one of the major challenges in the study of medical imaging.

While noise in medical images present a problem because they could mask and blur important but subtle features in the images, many proposed denoising techniques have their own problems. One of the widely discussed techniques is the wavelet thresholding scheme, which recognizes that by performing a wavelet transform of a noisy image, random noise will be represented principally as small coefficients in the high frequencies. Thus in theory a thresholding, by setting these small coefficients to zero, will eliminate much of the noise in the image. The *wavelet hard thresholding scheme*, which sets wavelet coefficients below certain threshold in magnitude to 0, is easy to implement and fast to perform, and depending on the threshold, it removes noise adequately. However, at the same time it also introduces artifacts as a result of the Gibbs oscillation near discontinuities. Since artifacts in medical images may lead to wrong diagnoses, the wavelet hard thresholding scheme is not practical for use in medical imaging without being combined with other techniques. An improvement over the wavelet hard thresholding is the *wavelet soft thresholding scheme* [1, 2], which significantly reduces the Gibbs oscillation but does not eliminate it. The effectiveness of wavelet thresholding schemes in general are limited with combining them with other techniques. These other more complex techniques often try to take account of geometric informations by using wavelet-like bases that better characterize discontinuities, such as curvelets [3, 4]. Nevertheless, they do not completely eliminate the Gibbs phenomenon. Other methods with varying success have also been studied by different authors, for example, [5–7].

Another approach employs variational principles and PDE-based techniques. In this approach, a noisy image is modeled as $z(x) = u_0(x) + n(x)$ where $u_0$ denotes the uncontaminated underlying image and $n$ denotes the noise. To reconstruct $u_0$ one considers the problem of minimizing

$$E(u) = \frac{\lambda}{2}\|u - z\|^2_{L^2(\omega)} + R(u), \tag{1}$$

where $\lambda > 0$, $\Omega$ is the domain on which $z$ is defined, and the term $R(u)$ is a regularization functional. Earlier efforts focused on least square-based functionals $R(u)$'s such as $\|\Delta\|^2_{L^2(\omega)}$, $\|\nabla u\|^2_{L^2(\omega)}$, and others. While noise can be effectively removed, these regularization functionals penalize discontinuity, resulting in soft and smooth reconstructed images, with subtle details lost. Again, for medical imaging this is not practical, as subtle details could very well yield crucial information about the patients.

A better choice for $R(u)$ was proposed in [8], in which $R(u)$ is the *total variation (TV)* of $u$ given by

$$R(u) = TV(u) := \int_{\Omega} |\nabla u| dx. \quad (2)$$

Intensive studies have shown that the total variation better preserves edges in $u$, thus it allows for sharper reconstructions, for example, [9–12]. Among all the PDE-based techniques, the TV minimization scheme is a candidate that offers the best combination of noise removal and feature preservation.

Solving the minimizers for the TV minimization (2), or (1) in general, amounts to solving certain PDEs, which is very similar to the anisotropic diffusion scheme proposed first in [13]. For the TV minimization it is easy to show that the PDE is given by

$$\nabla \cdot \left( \frac{\nabla u}{|\nabla u|} \right) - \lambda(u - z) = 0. \quad (3)$$

But in practice, one introduces the time variable $t$ and solves for $u(x, t)$ by time-marching the equation

$$u_t = \nabla \cdot \left( \frac{\nabla u}{|\nabla u|} \right) - \lambda(u - z), \quad u(x, 0) = z(x). \quad (4)$$

The end result $u(x, T)$, if $T$ is large enough, will have all noise removed. An important attribute of the TV minimization scheme is that it takes the geometric information of the original images into account, in that it preserves significant edges. In fact significant edges are sharpened. This is similar to the anisotropic diffusion methods see [13, 14] and references therein.

The time-marching of (4) is in essence solving for the minimizer of $E(u)$ by gradient flow. Two approaches are used for achieving the best combination of noise removal and feature preservation. The straighforward approach is to tune the parameter $\lambda$. Obviously if $\lambda$ is too large we may not remove enough noise. On the other hand, if $\lambda$ is too small it is well known that the scheme will remove too many features and end up with a cartoon-like piecewise constant image [15, 16]. But tuning the parameter $\lambda$ is time consuming. Since in practice there is no original image to compare to, and the assumption of i.i.d. Gaussian noise is not always realistic, tuning $\lambda$ often relies on experience and visual inspection. There is no automatic way for it as far as we know. A more widely used approach is to choose $\lambda$ in a reasonable range without being precise about the choice. Instead, we try to stop the time-marching before it reaches the ground state at a point that offers a good combination of noise removal and feature preservation. But again here we face the problem of deciding when to stop. There have been efforts in this direction, see, for example, [17, 18]. These proposed criteria are typically cumbersome and are based on some a priori knowledge about the noise such as the variance and type, which may not be realistic. With the explosion in volumes of medical images, this is a very significant issue.

In this paper we propose a *wavelet TV* denoising scheme. In our scheme, the wavelet coefficients are selected and modified subjecting to minimizing the TV norm of the reconstructed images. We demonstrate that while being as effective as the TV scheme in removing noise, the wavelet TV scheme allows us to modify the wavelet coefficients primarily in the high frequency domain, something that the regular TV scheme cannot do. Experiments show that the wavelet TV scheme preserves details like the regular TV scheme but offers a slightly higher PSNR in the reconstruction. It is also significantly faster in that far fewer iterations are needed for noise removal. The details of these improvements will be presented in a separate paper [19]. And unlike the traditional wavelet thresholding scheme, it does not introduce Gibbs' oscillations near discontinuities. These properties are consistent with other investigations that combine variational approaches with wavelet framework [20–24]. But more importantly, this scheme allows for an effective automatic stopping time criterion based on a certain statistical property of wavelet coefficients. An added advantage for our approach is that it leads to superior JPEG2000 compression for denoised images [21]. Given the increased use of JPEG2000 standard in medical imaging, this is a significant bonus.

### 2. THE WAVELET TOTAL VARIATION DENOISING METHOD

In this section, we describe our image denoising algorithm based on wavelet and TV minimization.

We start with a standard noisy monochromatic image model

$$z(x) = u_0(x) + n(x), \quad (5)$$

where $z(x)$, $u_0(x)$, and $n(x)$ are real valued functions defined on $\mathbb{R}^2$, and they are compactly supported since they represent images in our study. The function $u_0(x)$ denotes the underlying noise-free image, $z(x)$ the observed image, and $n(x)$ the noise. In our general model, we assume that $z(x)$, $u_0(x)$, and $n(x)$ are in some space of functions $\mathcal{F}$, such as $L^2(\Omega)$ for some domain $\Omega$. Let $\{\psi_j : j \in I\}$ be a basis for $\mathcal{F}$. This basis can be an orthonormal basis, such as wavelets [25, 26] if $\mathcal{F}$ is a Hilbert space, or any other type of bases in general. So for any $f(x) \in \mathcal{F}$ we have

$$f(x) = \sum_{j \in I} c_j \psi_j(x), \quad (6)$$

for some real $(c_j)$.

In [21] a wavelet TV minimization model is proposed, in which $\{\psi_j\}$ is taken to be a wavelet basis for $\mathcal{F} = L^2(\Omega)$. In that model, the wavelet coefficients are selected and modified to achieve the goals of image processing such as denoising and compression. In this paper, we refine the above model. Key to our innovation is an automatic stopping criterion, a feature we believe to be very important for medical applications. Another improvement is the multiscale fitting parameters targeting denoising in the high frequency domain, which yields a significant reduction in number of iterations needed to achieve the desired denoising as well as a small performance improvement in terms of PSNR on simulated noisy images.

We first describe the denoising part in the general setting. Let

$$z(x) = \sum_{j\in I} \alpha_j \psi_j(x) \tag{7}$$

and denote

$$u(x,\boldsymbol{\beta}) := \sum_{j\in I} \beta_j \psi_j(x), \tag{8}$$

where $\boldsymbol{\beta} = (\beta_j)$. Define the *total variation* functional by

$$F(u) := \int_{\mathbb{R}^2} |\nabla_x u(x,\boldsymbol{\beta})|\,dx + \frac{1}{2}\sum_{j\in I}\lambda_j(\beta_j - \alpha_j)^2, \tag{9}$$

where $u = u(x,\boldsymbol{\beta})$, $\lambda_j > 0$. In practice we often replace $|\nabla_x u(x,\boldsymbol{\beta})|$ by

$$|\nabla_x u|_\epsilon = \sqrt{|\nabla_x u|^2 + \epsilon}, \quad \text{with } 0 < \epsilon \ll 1. \tag{10}$$

The small parameter $\epsilon$ is used to prevent denominators from vanishing in numerical implementations. The goal of denoising is to minimize $F(u)$ and find the minimizer $u^* := u(x,\boldsymbol{\beta}^*)$ such that

$$F(u^*) = \min_{\boldsymbol{\beta}} F(u). \tag{11}$$

The objective functional in (9) differs somewhat from the one used in [21], where all $\lambda_j$'s are uniformly set to a single parameter $\lambda$. With uniform parameter $\lambda$ and an orthonormal basis $\{\psi_j\}$ the objective functional $F(u)$ is the same as the objective functional $E(u)$ in (1). Hence the minimizer of $F(u)$ would be the same as that of $E(u)$ for the regular TV scheme. By taking a basis that is not an orthonormal basis, such as a biorthogonal wavelet basis as we do in our implementation, $F(u)$ is typically not the same as $E(u)$, even with uniform parameter $\lambda_j$. With nonuniform $\lambda_j$'s the objective functional $F(u)$ can be significantly different from $E(u)$ in the original TV scheme. Like the regular TV denoising scheme, the wavelet TV scheme proposed here retain sharp edges without creating Gibbs' phenomenon.

One can use simple calculus of variation to obtain the derivative of the objective functional (9). For $u = u(x,\boldsymbol{\beta})$ where $\boldsymbol{\beta} = (\beta_j)$,

$$\begin{aligned}\frac{\partial F(u)}{\partial \beta_j} &= \int_{\mathbb{R}^2} \frac{\nabla_x u}{|\nabla_x u|}\cdot \nabla_x \psi_j dx + \lambda_j(\beta_j - \alpha_j)\\ &= -\int_{\mathbb{R}^2} \nabla_x \cdot \left[\frac{\nabla_x u}{|\nabla_x u|}\right]\psi_j dx + \lambda_j(\beta_j - \alpha_j).\end{aligned} \tag{12}$$

Then the Euler-Lagrange equation for the model is

$$-\int_{\mathbb{R}^2} \nabla_x \cdot \left(\frac{\nabla_x u}{|\nabla_x u|}\right)\psi_j(x)dx + \lambda_j(\beta_j - \alpha_j) = 0. \tag{13}$$

In practice, rather than solving the Euler-Lagrange equation (13) directly to denoise an image, we introduce an artificial time parameter $t$ and time-march the image using gradient flow. More precisely, we set $\boldsymbol{\beta} = \boldsymbol{\beta}(t) = (\beta_j(t))$ and solve the following time evolution equation:

$$\frac{\partial \beta_j}{\partial t} = \int_{\mathbb{R}^2} \nabla_x \cdot \left(\frac{\nabla_x u}{|\nabla_x u|}\right)\psi_j(x)dx - \lambda_j(\beta_j - \alpha_j), \quad \beta_j(0) = \alpha_j. \tag{14}$$

The minimizer of the TV wavelet model is the steady state of the above equation.

However, it is well known that TV minimization often leads to images with cartoonish features. More precisely, the denoising algorithm will remove noise as well as fine structures, such as textures and subtle details, from an image. The consequence is that unless the parameter $\lambda$ in (1) is carefully calibrated, if one evolves (14) for an extended time, the denoised image is often over-smoothed to the point that it is almost piecewise constant. The wavelet TV denoising scheme has the same issue. This is often unacceptable for most medical applications. In the original TV minimization scheme introduced in [8] or similar schemes such as anisotropic diffusion, there was no mechanism for stopping the time evolution. In fact, since the objective functionals do not measure information pertaining to noise in the processes, a mechanism to stop the time evolution automatically is virtually impossible. But in our wavelet TV denoising scheme this can be naturally done. The reason is that high frequency wavelet coefficients are well known to encode information about noise in images. This property of high frequency wavelet coefficients has served as the basis for virtually all wavelet denoising methods, such as the widely used hard or soft thresholdings, or wavelet shrinkage. Now, by choosing $\{\psi_j\}$ to be a wavelet basis, the same principle allows us to design a natural automatic stopping criterion for the wavelet TV minimization method, making it an extremely viable scheme for medical applications.

We now describe our automatic stopping criterion with the basis $\{\psi_j : j \in I\}$ being a wavelet basis—in our case we usually take the biorthogonal wavelet basis generated by the well-known 7–9 biorthogonal wavelets. (We remark that the conventional notation for wavelet bases use two or more indices, such as $\{\psi_{jk}\}$. In this paper we only use one index for conciseness, and there should not be any confusion). Like in the wavelet hard thresholding scheme, we first choose a threshold $\rho > 0$. Let $J_\rho = \{j \in I_D : |\beta_j(0)| = |\alpha_j| \le \rho\}$, where $I_D \subset I$ is the index set corresponding to the diagonal portion of the highest frequency wavelet coefficients. Intuitively speaking, as in the wavelet hard thresholding scheme, the coefficients $\{\beta_j(0) : j \in J_\rho\}$ will indicate how noisy the image is. In a noise-free image these wavelet coefficients will mostly be very close to 0. But in a noisy image they will be more substantial. Define $\mu(t) = (1/|J_\rho|)\sum_{j\in J_\rho}|\beta_j(t)|$. So $\mu(t)$ measures the noise in the image at time $t$. The key idea is that an automatic stopping criterion of the time evolution can be designed by measuring the reduction in the value $\mu(t)$ from the original value $\mu(0)$.

We can use two different approaches in setting the automatic stopping criterion. The first approach is the *relative criterion*. In the relative criterion, we consider $\mu(t)/\mu(0)$. We will stop the time evolution whenever this value goes below a threshold $b$. For example, we may set $b = 0.1$. This threshold

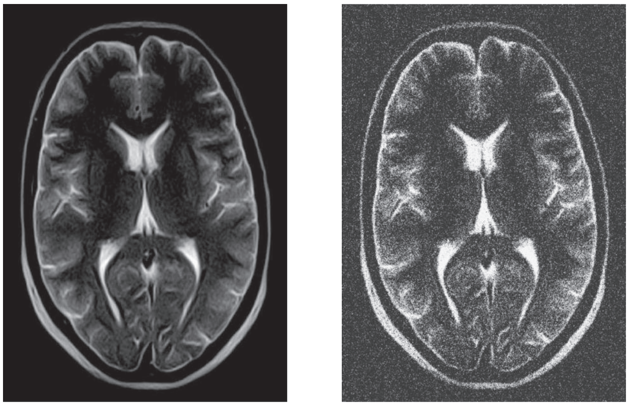

(a) (b)

Figure 1: (a) Original image. (b) Image with artificial additive Gaussian white noise, with PSNR = 2.55 (dB).

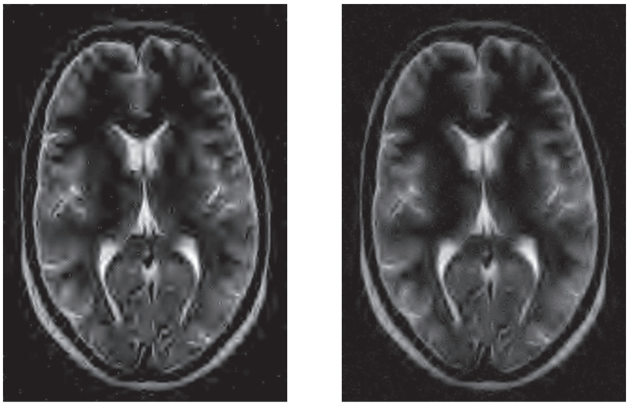

(a) (b)

Figure 2: (a) Denoised image by wavelet hard thresholding PSNR = 8.65 (dB), with the selected threshold that returns the best PSNR performance. (b) Denoised image by wavelet soft thresholding PSNR = 8.36 (dB); the threshold is selected to reach the best PSNR improvement. We note that the hard thresholding gives better PSNR performance because it is optimal in the $L^2$ norm sense, but the soft thresholding gives better visual quality because its Gibbs' oscillations are less severe.

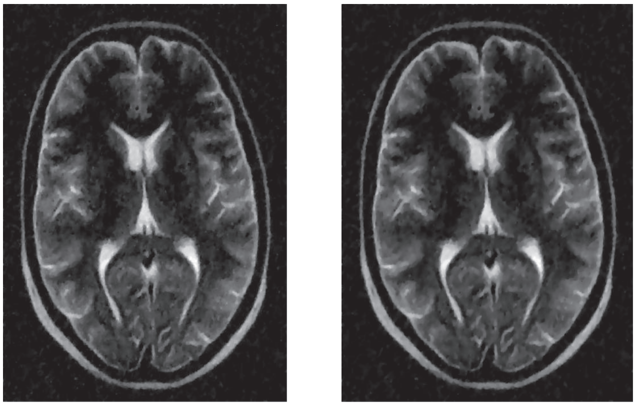

(a) (b)

Figure 3: (a) Denoised image by TV wavelet with fixed fitting parameter $\lambda_j$, the PSNR = 10.05 (dB). This image and the PSNR measurement are very similar to those of the regular TV scheme with the same parameter. (b) Denoised image by TV wavelet with variable fitting parameter $\lambda_j$ on different wavelet scales, the PSNR = 10.28 (dB).

intuitively says that we stop the time evolution when we have reduced noise by 90%. The second approach is the *absolute criterion*. In the absolute criterion, we stop the time evolution if $\mu(t)$ drops below a threshold $c$. Since in a noise-free image we expect $\mu(t)$ to be very close to zero, it is reasonable to set an absolute threshold for $\mu(t)$ to achieve a desired denoising effect.

In the actual implementation the value $\rho$ does not seem to affect the automatic stopping time sensitively. We usually take $\rho = (2/|I_D|)\sum_{j\in I_D}|\alpha_j|$. Both the relative criterion and the absolute criterion work well, although we typically use the relative criterion. For an image with moderate noise we set the threshold $b$ to be between 0.05 and 0.1. In the more noisy cases such as the images shown in this paper, we use smaller threshold $b$ around 0.03. We tested the automatic stopping time criterion on a number of MRI images for one lab. The thresholds for optimal performance stayed remarkably consistent. This is an important property for batch processing of medical images.

### 3. EXAMPLES

In this section we provide some examples to illustrate the performance of our algorithm. The first example is for testing. Artificial noise is added to an otherwise rather clean brain scan shown in Figure 1(a). The standard peak signal-to-noise ratio (PSNR) is employed to quantify the performance of denoising, where

$$\text{PSNR} = 10\log_{10}\left(\frac{255^2}{\|u-u_0\|_2^2}\right)\ (\text{dB}), \qquad (15)$$

where 255 is the maximum intensity value of the gray-scale images, $u_0$ the noise-free original image, $u$ the noise added image, and $\|\cdot\|_2$ the standard $L^2$ norm. A conventional criterion is that larger PSNR signifies better performance. In addition, we use visual inspection to compare the performance in preservation of edges and other geometric features, which is not reflected through the PSNR measurement. In all examples shown here, we use Daubechies 7–9 biorthogonal wavelets with symmetric extensions at the boundaries.

We performed denoising on the noise-added brain scan image using the standard wavelet thresholding schemes (Figure 2) and our wavelet TV schemes (Figure 3). The thresholds in the wavelet hard and soft thresholding were chosen after some trials to ensure the best performance (in terms of PSNR) for fairness. This actually exemplifies the problem we try to solve: the only way to get optimal result is through trial and error experiments with the threshold.

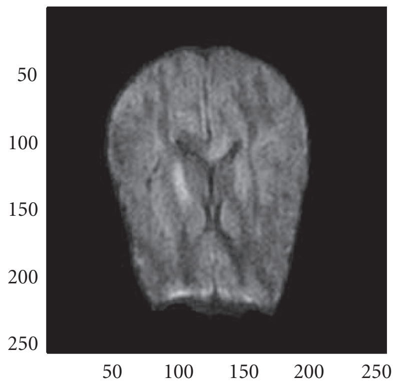

(a)

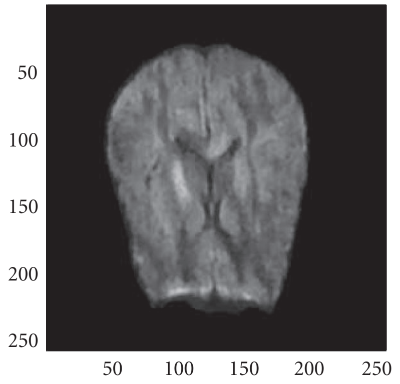

Figure 4: (a) Original image. (b) Denoised image using the TV wavelet algorithm.

For our wavelet TV scheme we use the relative approach and have set the autostopping threshold $b = 0.03$. We show results for two different choices of the parameters $\lambda_j$. In the first one we choose uniform $\lambda_j = 5$. In the second, the fitting parameters $\lambda_j$ for the coarsest level wavelet coefficients (including low frequencies) are all set to $\lambda_j = 400$. Afterwards with each finer level we decrease $\lambda_j$'s by a factor of 4. Similar idea of choosing the parameters has appeared in [27] for a different purpose. As one can see, the wavelet TV scheme in both examples outperforms the wavelet thresholding significantly. But more importantly, the wavelet TV image maintained sharpness and many fine details, while the wavelet thresholding image looks soft with details lost. The uniform fitting parameter example performed similarly to the regular TV scheme with the same parameter. The multiscale fitting parameters wavelet TV scheme has a small advantage in PSNR, and in our opinion is visually better. However, the number of iterations is significantly smaller than either the uniform $\lambda_j$ wavelet TV scheme or the regular TV scheme.

In the next example (Figure 4), we apply the algorithms with uniform $\lambda_j = 5$ to a real image without artificial noise. The original image appears quite noisy. We cannot judge the performance by examining the PSNR as we do not have a noise-free image with which we can compare. However, by visual inspection it is evident that the denoised image, while removing a substantial amount of noise, suffers virtually no degradation in sharpness and details.

### ACKNOWLEDGMENTS

We would like to thank Dr. Xiaoping Hu and his lab for providing us with the original image in Figure 2 and very helpful discussions. We would like to thank Dr. Guowei Wei for providing us with the image in Figure 1. Finally, we thank the anonymous referees for their valuable comments. The first author was supported in part by the National Science Foundation Grant no. DMS-0456538. The second author was supported in part by the National Science Foundation Grant no. DMS-0410062.

### REFERENCES

[1] D. L. Donoho, "De-noising by soft-thresholding," *IEEE Transactions on Information Theory*, vol. 41, no. 3, pp. 613–627, 1995.

[2] D. L. Donoho and J. M. Johnstone, "Ideal spatial adaptation by wavelet shrinkage," *Biometrika*, vol. 81, no. 3, pp. 425–455, 1994.

[3] E. J. Candès and D. Donoho, "Curvelets—a surprisingly effective nonadaptive representation for objects with edges," in *Curves and Surfaces*, A. Cohen, C. Rabut, and L. L. Schumaker, Eds., pp. 105–120, Vanderbilt University Press, Nashville, Tenn, USA, 1999.

[4] E. J. Candès and F. Guo, "Edge-preserving image reconstruction from noisy radon data," (Invited *special issue of the Journal of Signal Processing on Image and Video Coding Beyond Standards.*), 2001.

[5] A. Buades, B. Coll, and J.-M. Morel, "A non-local algorithm for image denoising," in *Proceedings of the IEEE Computer Society Conference on Computer Vision and Pattern Recognition (CVPR '05)*, vol. 2, pp. 60–65, San Diego, Calif, USA, June 2005.

[6] R. H. Chan, S. D. Riemenschneider, L. Shen, and Z. Shen, "Tight frame: an efficient way for high-resolution image reconstruction," *Applied and Computational Harmonic Analysis*, vol. 17, no. 1, pp. 91–115, 2004.

[7] J. Portilla, V. Strela, M. J. Wainwright, and E. P. Simoncelli, "Image denoising using scale mixtures of Gaussians in the wavelet domain," *IEEE Transactions on Image Processing*, vol. 12, no. 11, pp. 1338–1351, 2003.

[8] L. I. Rudin, S. Osher, and E. Fatemi, "Nonlinear total variation based noise removal algorithms," *Physica D*, vol. 60, no. 1–4, pp. 259–268, 1992.

[9] R. Acar and C. R. Vogel, "Analysis of total variation penalty methods for ill-posed problems," *Inverse Problems*, vol. 10, no. 6, pp. 1217–1229, 1994.

[10] A. Chambolle and P.-L. Lions, "Image recovery via total variation minimization and related problems," *Numerische Mathematik*, vol. 76, no. 2, pp. 167–188, 1997.

[11] T. F. Chan, S. Osher, and J. Shen, "The digital TV filter and nonlinear denoising," *IEEE Transactions on Image Processing*, vol. 10, no. 2, pp. 231–241, 2001.

[12] D. C. Dobson and C. R. Vogel, "Convergence of an iterative method for total variation denoising," *SIAM Journal on Numerical Analysis*, vol. 34, no. 5, pp. 1779–1791, 1997.

[13] P. Perona and J. Malik, "Scale-space and edge detection using anisotropic diffusion," *IEEE Transactions on Pattern Analysis and Machine Intelligence*, vol. 12, no. 7, pp. 629–639, 1990.

[14] Y. Sun, P. Wu, G. W. Wei, and G. Wang, "Evolution-operator-based single-step method for image processing," *International Journal of Biomedical Imaging*, vol. 2006, Article ID 83847, pp. 27 pages, 2006.

[15] Y. Meyer, *Oscillating Patterns in Image Processing and Nonlinear Evolution Equations*, vol. 22 of *University Lecture Series*, American Mathematical Society, Providence, RI, USA, 2001.

[16] S. Osher, A. Solé, and L. Vese, "Image decomposition and restoration using total variation minimization and $H^1$," *Multiscale Modeling and Simulation*, vol. 1, no. 3, pp. 349–370, 2003.

[17] P. Mrázek and M. Navara, "Selection of optimal stopping time for nonlinear diffusion filtering," *International Journal of Computer Vision*, vol. 52, no. 2-3, pp. 189–203, 2003.

[18] J. Weickert, "Coherence-enhancing diffusion of colour images," *Image and Vision Computing*, vol. 17, no. 3-4, pp. 201–212, 1999.

[19] Y. Wang and H.-M. Zhou, "Denoising natural color images," preprint.

[20] A. Chambolle, R. A. DeVore, N.-Y. Lee, and B. J. Lucier, "Nonlinear wavelet image processing: variational problems, compression, and noise removal through wavelet shrinkage," *IEEE Transactions on Image Processing*, vol. 7, no. 3, pp. 319–335, 1998.

[21] T. F. Chan and H. Zhou, "Optimal constructions of wavelet coefficients using total variation regularization in image compression," UCLA CAM Report 00-27, Department of Mathematics, UCLA, Los Angeles, Calif, USA, July 2000.

[22] T. F. Chan and H. Zhou, "Total variation wavelet thresholding," submitted to *Journal of Computational Physics*.

[23] S. Durand and J. Froment, "Artifact free signal denoising with wavelets," in *Proceedings of IEEE International Conference on Acoustics, Speech and Signal Processing (ICASSP '01)*, vol. 6, pp. 3685–3688, Salt Lake City, Utah, USA, May 2001.

[24] F. Malgouyres, "Mathematical analysis of a model which combines total variation and wavelet for image restoration," *Journal of Information Processes*, vol. 2, no. 1, pp. 1–10, 2002.

[25] I. Daubechies, *Ten Lectures on Wavelets*, SIAM, Philadelphia, Pa, USA, 1992.

[26] G. Strang and T. Nguyen, *Wavelets and Filter Banks*, Wellesley-Cambridge Press, Wellesley, Mass, USA, 1996.

[27] Y. Lu, L. Shen, and Y. Xu, "Shadow block iteration for solving linear systems obtained from wavelet transforms," *Applied and Computational Harmonic Analysis*, vol. 19, no. 3, pp. 359–385, 2005.

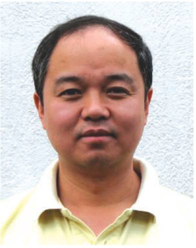

**Yang Wang** graduated from the University of Science and Technology of China in 1983 with B.S. degree in mathematics, and received his doctorate degree in mathematics from Harvard University in 1990 under the guidance of David Mumford. He has been a Faculty Member at Georgia Tech since, and is currently the Asscoiate Chair and the Undergraduate Coordinator of Mathematics. His research interests include wavelets and applications, tiling, fractal geometry and applications, image processing, among others.

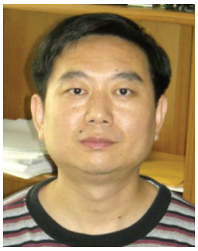

**Haomin Zhou** graduated from the Peking University in 1991 with B.S. degree in mathematics. He received his M. Phil. degree in applied mathematics from the Chinese University of Hong Kong in 1996 and his Ph.D. degree in mathematics from University of California, Los Angeles, in 2000. He spent 3 years in California Institute of Technology as a Postdoctoral Researcher before he became a Faculty Member of Georgia Institute of Technology, in 2003. His research interests include wavelets and applications, numerical PDEs, image processing, as well as stochastic differential equations and applications.

